# Surgical risk of CSF leakage following endoscopic transorbital approach for anterior and middle skull base pathologies: a systematic review and meta-analysis

**DOI:** 10.1007/s10143-025-03426-z

**Published:** 2025-03-06

**Authors:** Sergio Corvino, Jacopo Berardinelli, Giuseppe Corazzelli, Roberto Altieri, Iacopo Dallan, Francesco Corrivetti, Matteo de Notaris

**Affiliations:** 1 Department of Neurosciences, Reproductive and Odontostomatological Sciences, Neurosurgical Clinic, School of Medicine, University of Naples “Federico II”, 80131 Naples, Italy; 2 Multidisciplinary Department of Medical-Surgical and Dental Specialties, University of Campania “Luigi Vanvitelli”, Naples, Italy; 3 Skull-Base and Rhino-Orbital Surgery Unit, Azienda Ospedaliero-Universitaria Pisana, Pisa, Italy; 4 Department of Neurosurgery, A.O.U. “San Giovanni di Dio e Ruggi d’Aragona”, Salerno, Italy; 5 Department of Neurosciences, Reproductive and Odontostomatological Sciences, Division of Neurosurgery, University of Naples Federico II, 80131 Naples, Italy

**Keywords:** Endoscopic transorbital approach, CSF leak, Skull base reconstruction, Complications, Outcome

## Abstract

The endoscopic superior eyelid transorbital approach (SETOA) has demonstrated considerable versatility and effectiveness in managing various paramedian anterior and middle skull base pathologies. However, as with any relatively new technique, potential complications remain. We conducted an extensive literature search in MEDLINE and Embase in accordance with PRISMA guidelines including case reports and surgical series reporting cerebrospinal fluid (CSF) leak rate following SETOA for intracranial pathologies. Factors analyzed included lesion location (extra- or intra-axial), reconstruction techniques, and complication management. ROBINS-I tool was employed to assess the risk of bias. Twenty-five studies including 240 cases were eligible. The majority of lesions were intradural extra-axial (68.3%), while trigeminal schwannomas comprised all extradural cases (25.0%). Sixteen patients (6.6%) presented intradural intra-axial tumors. Osteodural reconstruction involved dural substitutes in one third of the cases (32.5%) either alone (14.2%) or combined with fat free graft (18.3%). CSF leak occurred in 6 patients (2.50%), mostly resolving via conservative management (66.6%). The risk of postoperative CSF leak was found to be significantly higher in patients undergoing resection for intra-axial tumors (OR 0.13, 95% CI: 0.04–0.49) compared to those undergoing resection for extra-axial (OR 0.01, 95% CI: 0.00–0.02; I^2^ = 0%; *p* < 0.001). Key limitations include the retrospective nature and small sample sizes among included studies as well as data heterogeneity and lack of standardized protocols for reconstruction across studies. SETOA appears safe for addressing selected extradural and intradural skull base pathologies with a low postoperative CSF leak rate. The natural repositioning of the orbital content to its original position may be instrumental in preventing its postoperative occurrence. The investigation followed a prespecified protocol registered on PROSPERO (PROSPERO 2024 CRD42024614111).

**Supplementary Information:**

The online version contains supplementary material available at 10.1007/s10143-025-03426-z.

## Clinical trial number

Not applicable.

## Introduction

Over the last two decades, the indications of the endoscopic superior eyelid transorbital approach (SETOA), both in standard and extended variations, for the management of several neoplastic, vascular, functional and traumatic neurosurgical pathologies, mainly affecting the paramedian anterior and middle skull base, have rapidly increased thanks to its peculiar features, including the minimally invasive nature, short distance and direct access to the target, reduced bone destruction, minimal brain retraction and manipulation, satisfactory esthetic result, short hospital-stay, and rapid patient recovery [1–14].

Nevertheless, despite these benefits, as any other surgical approach, especially if recently introduced, it is not free from potential complications [2, 14–16], which can be classified into three groups: those associated to the lesion exposure, those associated to the lesion removal and those associated to the reconstruction of the osteo-dural defect.

The first ones derive from the creation of the surgical pathway to the target, thus can occur from the skin to the extra- or intradural stage, and include eyelid swelling, ptosis, mechanical sores or electrical burns on the skin, ocular disfunction, superficial punctate keratitis, wound infection and/or necrosis [2, 15, 17]. The second group of complications is related to the manipulation of the lesion and adjacent neurovascular structures, and include nervous (i.e. brain edema, seizures, sensory-motor deficit along the territory of distribution of a cranial nerve) and vascular (hemorrhagic infarction of surgical field, cerebral ischemia) damages. Finally, complications related to the reconstruction of the osteo-dural defect which include cerebrospinal fluid fistula (CSF leak), meningoencephalocele and enophthalmos. While complications belonging to the second group are mainly related to the lesion features, such as its size, pattern of growth, consistency, and the surgical technique and instruments required for its removal, those ones belonging to the first and third groups are mainly related to the surgical procedure, therefore any effort should be attempted to minimize or nullify them.

Most of these peri- and postoperative complications are transient, such as eyelid and periorbital edema, diplopia [15], CSF leak [2]; rarer reported persistent complications include gaze palsy or ocular pain [15], persistent vision loss [18], abducens nerve paresis [19], postoperative facial numbness [20], which must be carefully considered due to their significant impact on postoperative and long-term patient satisfaction. Their occurrence is related to the patient and lesion features, as well as on the surgeon expertise.

In this setting, special attention should be reserved to the CSF leak, which can potentially account for severe morbidity, including meningitis, brain herniation and tension pneumocephalus, up to death of patients, and which can be prevented with a meticulous watertight closure of the osteo-dural defect of the skull base.

The present systematic literature review investigates the rate of CSF leak after endoscopic transorbital approach for intracranial pathologies, based on the extra- or intra-axial nature of the lesion, the goal of surgery, the reconstruction technique and the management adopted with the aim to identify potential risk factors for CSF leak, discuss the clinical implication and provide recommendations for the surgical practice.

## Methods

### Search strategy

A Medline search from 2012 to September 2024 on MEDLINE and Embase online electronic databases was made in accordance with Preferred Reporting Items for Systematic Reviews and Meta-Analysis (PRISMA) guidelines [21], by using the following key phrases: (“endoscopic transorbital approach” OR “transorbital surgery”) AND (“intracranial intradural pathologies” OR “intracranial intra-axial pathologies” OR “intracranial extradural pathologies” OR “intracranial extra-axial pathologies”) AND (“CSF leak” OR “complications” OR “outcome”). Full search strategy is fully presented in Online Resources 1.

### Inclusion and exclusion criteria

**Fig. 1:**
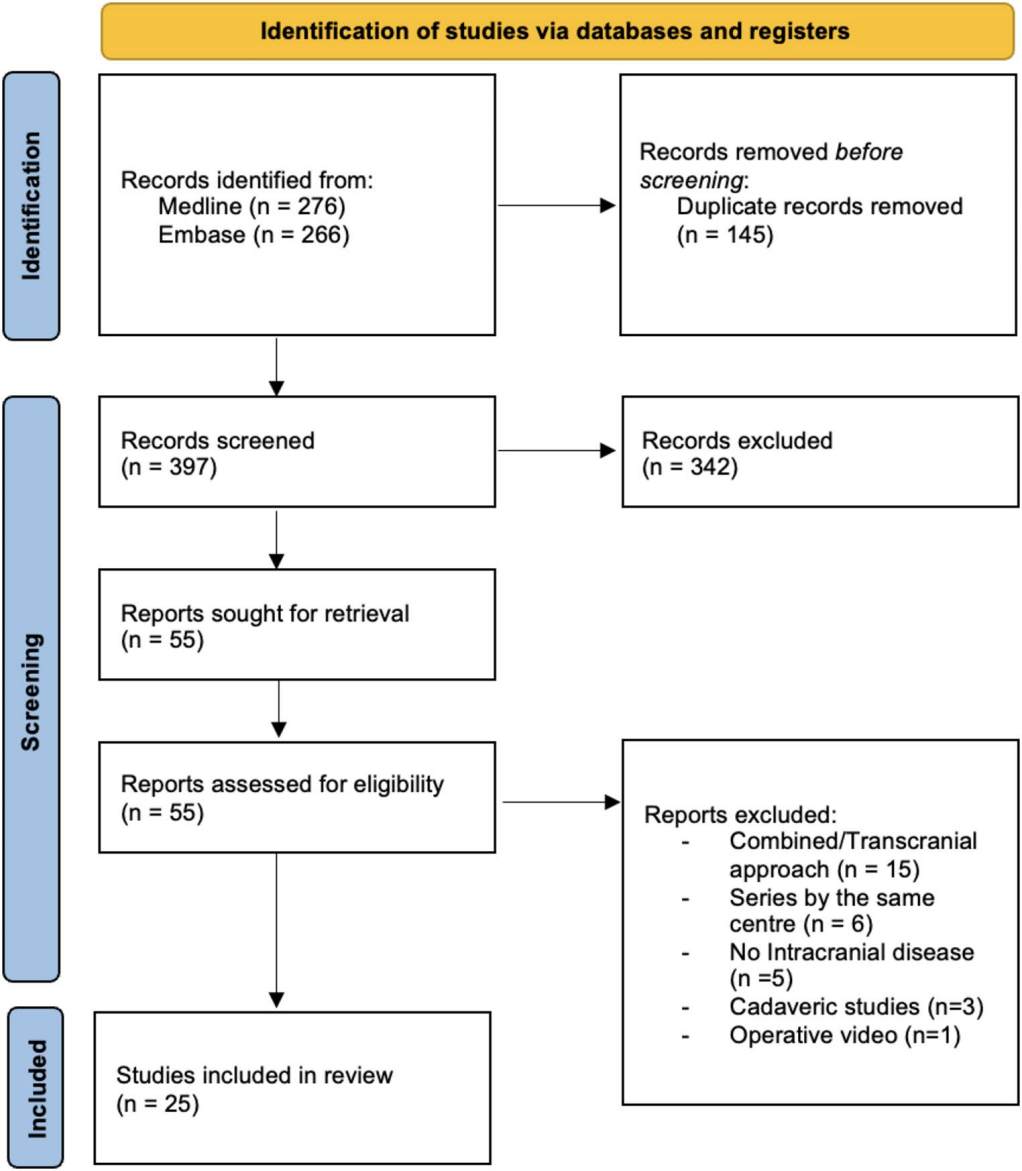
Flow chart showing the methods for the selection of the studies included in the review

The inclusion criteria were surgical series and case reports reporting the rate of CSF leaks after endoscopic transorbital approaches for intracranial extra- or intra-axial pathologies. Studies with non-English abstracts were excluded. Additionally, studies depicting combined approaches were excluded unless specific data for transorbital approaches could be uniquely extracted. Cadaveric studies, reviews, isolated endoscopic endonasal approaches, and/or isolated open transcranial approaches were also excluded. After duplicates removal, all abstracts were evaluated, and each article of interest was marked for further review. The full text of the marked studies was screened by two authors independently (S.C. and J.B.) and included in this systematic review following inclusion and exclusion criteria, as summarized in Fig. 1. Reason for exclusion of studies during the eligibility screening process is presented in Online Resources 1. Reports included were classified for authors, year and design of study; analyzed factors included nature of pathology (extradural, intradural extra-axial, intra-axial), number of cases, goal of surgery, extent of resection, type of osteo-dural defect reconstruction. postoperative CSF leak occurrence and management as well as intra and postoperative complications.

### Risk of bias assessment

ROBINS-I tool was applied to evaluate the Risk of Bias (RoB) in non-randomized controlled trials (non-RCTs), detected by the screening process (Online Resources 1). The overall RoB was classified as follows: critical, serious, moderate, low, or no information. The two independent investigators (S.C. and J.B.) separately assessed the RoB. A third author (M.d.N.) was consulted to resolve any disagreement between investigators.

### Statistical analysis

Data were collected through an extensive examination of patients described in the literature. The raw data were entered into Microsoft Excel (version 16.91 for Mac) for preliminary organization. Statistical analysis was conducted using R (version 4.0.2, The R Foundation for Statistical Computing) and RStudio (version 1.2.1335, posit.co). A p-value < 0.05 was considered statistically significant.

Data regarding CSF leak rates, tumor characteristics (extra-axial vs. intra-axial), and type of reconstruction (use of dural substitutes, autologous flaps, or implementation of fat free grafts) were aggregated and analyzed. Odds ratios (ORs) for postoperative CSF leak rates were calculated using the “metabin” function in R. The choice between random-effects and fixed-effects models was guided by I^2^ values, as recommended by standard statistical practice. Forest plots with subgroup analyses were generated using the “forest” function in R.

Heterogeneity was formally assessed using Q, I^2^, and τ^2^ statistics. Following the Cochrane Statistics Methods Group guidelines, I^2^ values were interpreted as follows: 0–40% (low heterogeneity), 30–60% (moderate heterogeneity), 50–90% (substantial heterogeneity), and 75–100% (considerable heterogeneity).

## Results

### Literature review

A comprehensive systematic literature review disclosed 542 studies. After duplicates removal, screening full texts of the marked studies included according to the inclusion criteria, 55 studies were identified. After removing the reports including series by the same center, series reporting aggregate data on combined approaches, cases without intracranial space involvement, cadaveric studies and operative videos, 25 studies [6, 12, 22–44] including 240 cases were eligible for the review (Table 1).

**Table 1:** Summary of included studies reporting endoscopic transorbital resection of intracranial tumors and postoperative CSF leak rates. CS: cavernous sinus, CSF: cerebrospinal fluid, PitNET: pituitary neuroendocrine tumor, SFT: solitary fibrous tumor, VP: Ventricolo-peritoneal

Authors and year	*N*. of cases	Pathology	Goal of surgery	Reconstruction	Csf leak, *n* (%)	Management
Chen et al. [22] 2015	2	Amygdalo hippocampectomy	Lesionectomy	Dural approximation with sutures, free local tissue graft, dural sealant, tailored nylon sheet.	1 (50)	VP Shunt
Kong et al. [23] 2018	4	Sebaceus gland carcinoma (1) Cystic teratoma (1) Plasmacytoma (1) Metastatic osteosarcoma (1)	Resection	Intra and extradural fascia lata/dural substitute.	0 (0)	
De Rosa et al. [24] 2019	1	Spheno-orbital meningioma	Debulking	Dural substitute, fibrin glue, Extradural fat free graft.	0 (0)	-
Jeon et al. [12] 2018	7	Trigeminal schwannoma (3) Meningioma (2) Dermoid cyst (1) Metastasis (1)	Resection (6) Debulking (1)	Dural substitute/Fascia lata	0 (0)	
In Woo et al.(25) 2021	17	Spheno-orbital meningioma	Resection	Intra and extradural fat free graft, Intra and extradural fascia lata	1 (5.9%)	Surgical repair
Kong et al. [26] 2020	41	Spheno-orbital meningioma	Resection	Intra and extradural fat free graft, Intra and extradural fascia lata; titanium mesh.	2 (4.9)	Lumbar drainage
Lim et al. [27] 2020	3	Trigeminal schwannoma (2) Sphenoid wing meningioma (1)	Resection	-	0 (0)	
Kim et al. [28] 2021	1	Insular glioma	Resection	Dural substitutes, fibrin glue, porous polyethylene implant.	0 (0)	
Park et al. [29] 2021	7	Diffuse astrocytoma (3) Anaplastic Astrocytoma (3) Glioblastoma (1)	Resection	Intra and extradural fascia lata/dural substitute.	0 (0)	
Lim et al. [30] 2022	7	Clinoid meningioma (6) CS Meningioma (1)	Resection	Fat free graft/Muscle pad/Dural substitute	0 (0)	
Foulsham et al. [31] 2022	1	Sphenoid wing meningioma	Resection	Intra and extradural dural substitute.	0 (0)	-
Corvino et al. [2] 2022	1	Spheno-orbital meningioma	Resection	Fat free graft Fascia lata	0 (0)	-
Locatelli et al. [33] 2022	13	Spheno-orbital meningioma	Resection	Dural substitute, fibrin glue, extradural fat graft.	0 (0)	
Bander et al. [34] 2023	5	Dermoid Trigeminal schwannoma PitNET SFT Craniopharyngioma	Debulking (4)	-	0 (0)	-
Han et al. [35] 2023	8	Spheno-orbital meningioma (5) CS Meningioma (1) Trigeminal schwannoma (1) Epidermoid cyst (1)	Resection (7) Biopsy (1)	Fascia lata, extradural fat free graft, titanium mesh.	0 (0)	
de Notaris et al. [6] 2023	1	Pharingeal Carcinoma Metastasis	Biopsy	Intra and extradural fat free graft Dural substitutes	0 (0)	
Kong et al. [45] 2023	50	Trigeminal schwannoma	Resection	Intra and extradural fascia lata/dural substitute.	0 (0)	
Polster et al. [37] 2023	1	Melanoma	Resection	Dural substitute, fat graft	0 (0)	
Serioli et al. [38] 2023	3	Sphenoid wing meningioma	Resection	Intra and extradural fat free graft, Intra and extradural dural substitute; fibrin glue	0 (0)	
Mathios et al. [39] 2024	17	Spheno-orbital meningioma (6) Sphenoid wing meningioma (2) Middle fossa meningiomas (2) Trigeminal Schwannoma (2) Epidermoid (1) Dermoid (1) PitNET (1) Inflammatory mass (1) NSCLC metastasis (1)	Resection	Dural substitute Fat graft	1 (5.9)	Lumbar drainage
Nishijima et al. [40] 2024	1	Temporal cavernous malformation	Resection	-	0 (0)	
Karımzada et al. [41] 2024	32	Sphenoid wing meningioma	Resection	Dural substitutes	0 (0)	
Carnevale et al. [42] 2024	8	Sphenoid wing meningioma (6) Middle fossa meningiomas (2)	Resection	Dural substitute, fat graft, fibrin glue.	1 (13)	Lumbar drainage
Corrivetti et al. [43] 2024	1	Trigeminal schwannoma	Resection	-	0 (0)	-
Di Somma et al. [15] 2024	8	Spheno-orbital meningioma (5) Intradiploic meningioma (1) Temporal pole arachnoid cyst (1) CS Hemangioma (1)	-	Intra and extradural fat free graft Dural substitutes Fascia lata	0 (0)	-

### Pathological data

Tumor volumes was inconsistently documented among different studies and reported in less than half of the study sample (100/240; 41.7%) attesting at a mean of 12.55 cm^3^.

**Fig. 2:**
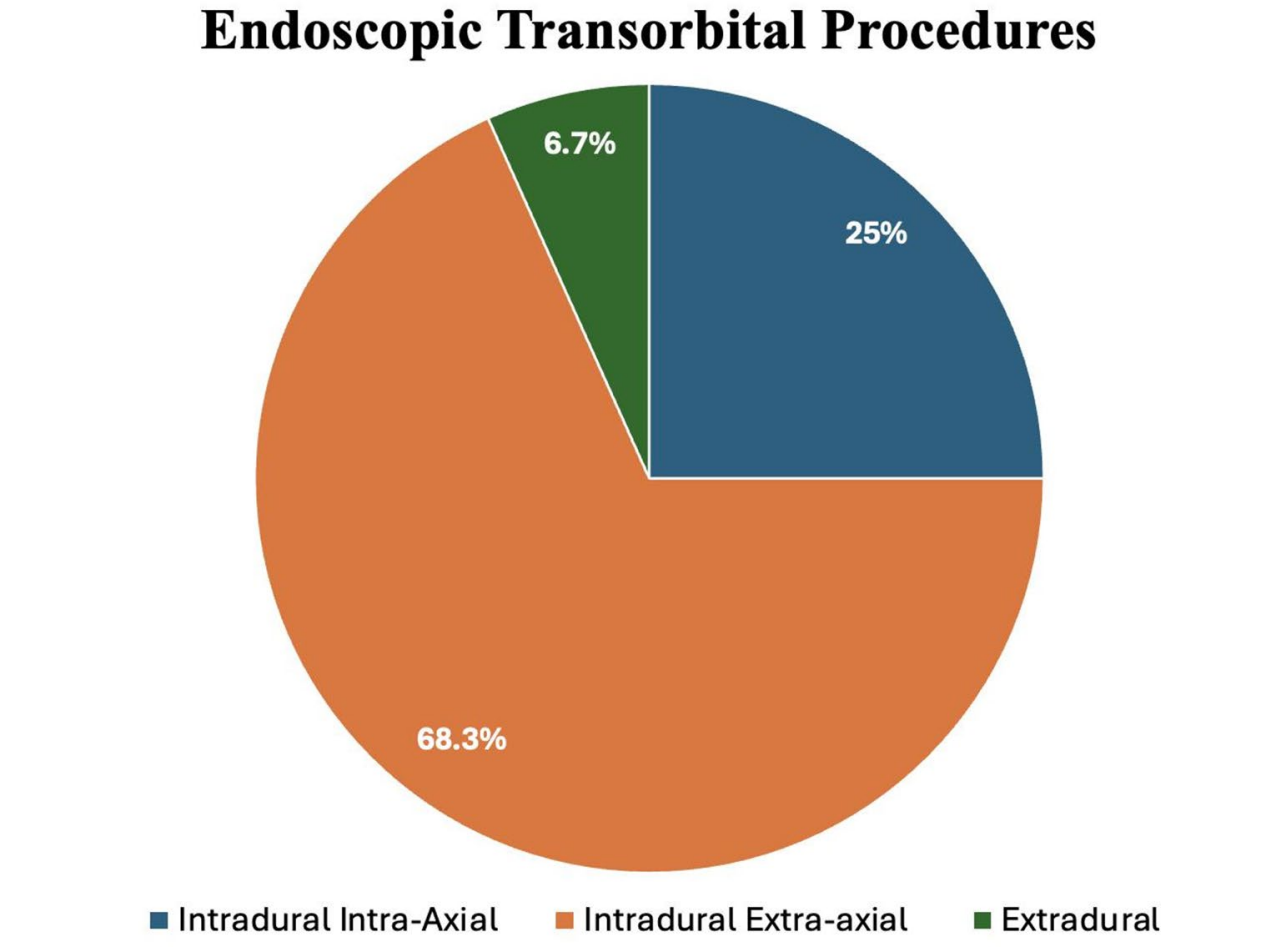
Distribution of Pathologies Treated with Endoscopic Transorbital Procedures according to the relationship with dura mater

Most lesions were intradural extra-axial (n.=164/240, 68.3%) and were mainly represented by meningiomas (n.=149/240; 62.1%), especially spheno-orbital meningiomas (89/240; 37.1%). Extradural lesions were exclusively represented by trigeminal schwannomas (n.=60/240; 25.0%). Finally, only 16 patients were affected by intradural intra-axial tumors (n.=16/240; 6.6%), mostly belonging to the glial series (n.=8/240; 3.3%) or metastatic tumors (n.=5/240; 2.1%). Frequency of distribution of pathologies treated by SETOA according to the relationship with dura mater is shown in Fig. 2.

Goal of surgery was resection in most of the studied sample with only 8 patients undergoing TOA for tumor biopsy (8/240; 3.3%). GTR/NTR were achieved for 135 patients (56.3%) while the remaining cases underwent a STR (95/240; 39.6%). Among the included studies, data on the extension of the approach through a lateral rim osteotomy were available for a subset of 199 patients, of whom 49 (24.6%) underwent an extended transorbital procedure. Surgery goals also involved orbital wall hyperostosis decompression for 41 patients harboring a sphenoid wing meningioma.

### Reconstruction

Reconstruction of skull base defect was clearly stated in 21 studies comprising a total of 230 patients (n.=230/240; 95.8%).

**Fig. 3:**
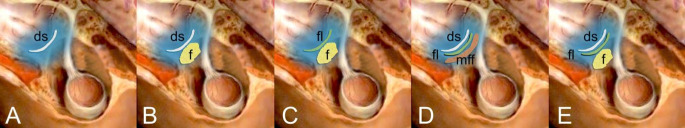
Schematic illustration depticting various applied technique of dural reconstruction after TOA. Dural substitues (ds) is the most common material used for dural reconstruction and can be implemented or substitute with free autologous tissue flaps such as fascia lata (fl.) or muscular free flap (mff). Intradural fat free flap (f) can also be added both to support dural reconstruction and to fill postoperative surgical empty space. **ds**: Dural substitute; **f**: Fat free flap; fl.: Fascia lata; **mff**: Muscle free flap

Dural substitutes were used in almost half of the cases (n.=78/240; 32.5%) either alone (n.=34/240; 14.2%) or in combination with autologous fat free graft (n.=44/240; 18.3%). Fascia lata and fat grafts were another commonly used reconstruction approach (n.=59/240; 24.6%). In 83 cases either dural substitutes and/or fascia lata free flap were reported as material used for dural reconstruction (n.=83/240; 34.6%). No significant differences were found in the type of reconstruction used after resection of extra and intra axial lesions. Frequency of different reconstruction solutions are shown in Table 2; Fig. 3.

**Table 2:** Different reconstruction strategies applied after endoscopic transorbital approaches and relative frequency and rate of CSF leak

Reconstruction	Cases, *n*	CSF leak	Rate	Pathology
Dural substitutes	34	0	-	
Dural substitutes and fat	44	2	4.54%	2 SW
Fascia lata and fat	59	2	3.39%	2 SOM
Dural substitutes/Fascia lata/Muscle free flap	83	1	1.20%	1 SOM
Dural substitutes/Fascia lata/Fat	8	0	-	
Free local flap	2	1	50.0%	1 AH
Nd	10	0	-	

**SOM**: Spheno-Orbital Meningiomas; **SWM**: Sphenoid-Wing Meningiomas; **AH**: Amygdalohippocampectomy

### CSF leak rate

Among the overall sample postoperative CSF leak rate was documented in 6 patients, five of which followed surgical resection of a spheno-orbital (n.=3/106; 2.83%) or a sphenoid wing (n.=2/45; 4.44%) meningioma and one following an endoscopic amygdalohippocampectomy. The cumulative CSF leak rate attested at 2.5% (95% C.I. 1.1–5.4).

In most cases (n.=4/6; 66.6%) CSF leak resolved with conservative management via placement of a lumbar drain resulting in a total CSF leak rate without the need for revision surgery of 0.96%. In the remaining cases it was managed with placement of a ventricular-peritoneal shunt (n.=1/6; 16.7%) or with direct surgical repair (n.=1/6; 16.7%).

CSF leak rate after resection of extradural, intradural extra-axial and intra-axial lesions attested at 0%, 3.05% and 6.25%, respectively.

**Fig. 4:**
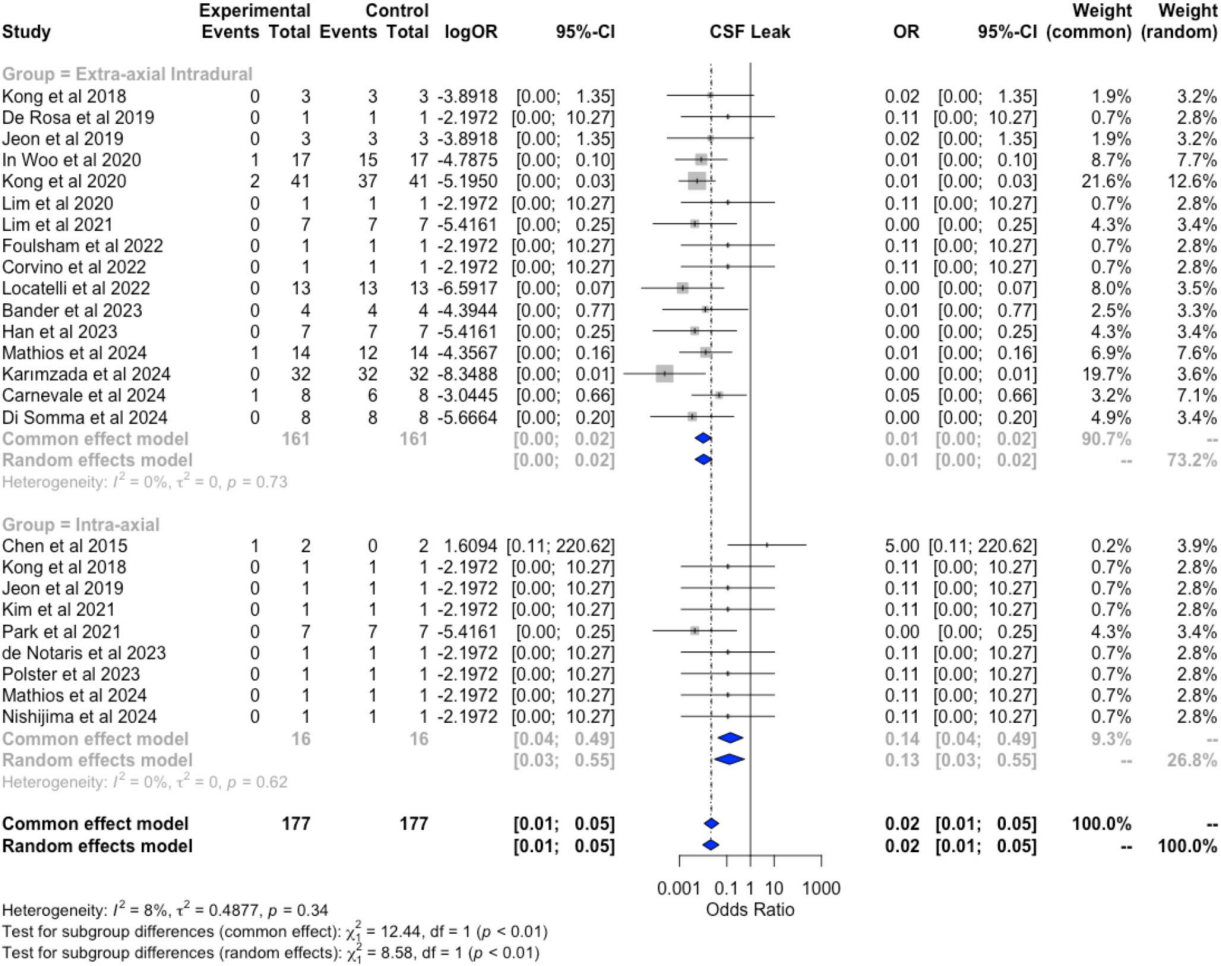
Pooled analysis and comparison revealed an increased odds ratio for postoperative CSF leaks following resection of intradural extra-axial tumors compared to extra-axial tumors (*p* < 0.001), with significantly low heterogeneity among the study sample (I^2^ = 8%)

The risk of postoperative CSF leak was found to be significantly higher in patients undergoing resection for intra-axial tumors (OR 0.13, 95% CI: 0.04–0.49; I^2^ = 0%) compared to those undergoing resection for extra-axial tumors (OR 0.01, 95% CI: 0.00–0.02; I^2^ = 0%; *p* < 0.001) or for the subgroup of intradural extra-axial tumors (OR 0.01, 95% CI: 0.00–0.02; I^2^ = 0%; *p* < 0.001) (Fig. 4).

The use of either a dural substitute or an autologous fascia lata free flap did not significantly affect the risk of postoperative CSF leaks (*p* = 0.47). Similarly, no significant differences in postoperative risk were observed between patients undergoing reconstruction with or without a fat free graft (*p* = 0.17). No cases of postoperative CSF fistula have been reported following the use of an extended transorbital approach.

Frequencies of different pathologies treated via TOA, along with their relative CSF leak rates, are shown in Table 3, while forest plots for the pooled subgroup analysis are presented in Fig. 4 and Figure OR3.

**Table 3:** Pathologies treated via an endoscopic transorbital approach and relative frequency and rate of CSF leak. No difference was found when comparing CSF leak rate for intra and extra axial tumors resection

EXTRA DURAL LESIONS			
	Cases, *n*	CSF leak	Rate
**Trigeminal Schwannomas**	**60**	**0**	-

INTRADURAL EXTRA-AXIAL LESIONS			
	Cases, n	CSF leak	Rate
**Meningiomas**	149	5	3.35%
Spheno-Orbital	89	3	3.37%
Sphenoid Wing	45	2	4.44%
Cavernous sinus	2	0	-
Clinoid	6	0	-
Middle Fossa	4	0	-
Nd	3	0	-
**Other intra-dural lesion**	15	0	-
**TOTAL**	**164**	**5**	**3.05%**

INTRADURAL INTRA-AXIAL LESIONS			
	Cases, n	CSF leak	Rate
**Gliomas**	8	0	-
**Metastasis**	5	0	-
**Other**	3	1	33.33%
**TOTAL**	**16**	**1**	**6.25%**

### Early and long-term postoperative complications

Follow-up data were reported for 205 patients (205/240, 85.4%) with a mean duration of 17.8 months.

Ocular complications were consistently documented across the study sample. Diplopia (17/240, 7.1%) and ptosis (16/240, 6.7%) were common early postoperative complications; however, most cases were self-resolving, with only 1 case (1/240, 0.4%) of long-term diplopia and 4 cases (4/240, 1.7%) of mild to moderate ptosis. Postoperative visual worsening was reported in 8 cases (8/240, 3.3%), 3 of which were permanent and associated with macular hemorrhage requiring vitrectomy, development of Terson syndrome, and a macular hemorrhage (3/240, 1.2%). Episodic complications included a self-resolving pulsatile exophthalmos and a case of transient esotropia.

Postoperative eyelid edema and conjunctival chemosis were noted in 31 (31/240, 12.9%) and 2 (2/240, 0.8%) patients, respectively, with resolution in all cases during the follow-up period. The most frequent postoperative complication was trigeminal dysesthesia, reported in 27 cases (27/240, 11.3%), with 21 cases (21/240, 8.8%) persisting at the last clinical follow-up.

Finally, two cases of intraoperative ICA injury and one case of postoperative surgical bed hematoma requiring surgical evacuation were reported among the included studies.

## Discussion

SETOA, when applied to selected intracranial pathologies affecting the paramedian anterior and middle skull base, has shown promising surgical, functional, and aesthetic outcomes [2, 23]. Its increasing indications and the growing number of surgical series addressing a variety of pathologies [6, 9, 11, 12, 26, 39, 46] highlight its potential as a minimally invasive alternative to transcranial approaches in specific scenarios.

For example, in spheno-orbital meningiomas, particularly in cases involving the lateral orbital compartment with proptosis as the primary symptom, the TOA provides a less invasive yet effective surgical option. Conversely, its anatomical constraints make it less suitable for large intracranial lesions with a medial extension toward the sellar region [26, 33]. In such cases, more traditional transcranial approaches remain the preferred choice.

When comparing the TOA to transcranial methods, one notable advantage is its lateral-to-medial trajectory for accessing medial temporal lobe lesions. This trajectory runs parallel to the hippocampus, allowing for a shorter surgical pathway without requiring temporal lobe retraction or lateral neocortex disruption, as is often necessary in transcranial approaches [22, 28]. However, the TOA still poses challenges, including achieving precise bimanual dissection, controlling bleeding, and avoiding orbital injury. These technical hurdles highlight the need for ongoing refinements before the ETOA can be standardized as a safe and practical option.

A critical step in standardizing this technique is the evaluation of potential postoperative complications, particularly CSF leaks, which are a significant concern in skull base surgery. CSF leaks represent one of the main concerns in skull base surgery, including the transorbital route, and it should be considered to avoid further potential severe complications. Several factors have been associated to the risk of postoperative CSF leakage after endoscopic endonasal (EEA) and transcranial skull base techniques, including body mass index, multiple operations, tumor size, tumor invasion, hard texture, and intraoperative cerebrospinal fluid leakage [47, 48]. In EEA, the use of a pedicled vascularized flap is associated with reduced risk of a CSF leak, particularly in overweight patients [48]. Therefore, a careful and meticulous reconstruction of the bony or osteo-dural defect aiming a watertight closure is mandatory.

In this context, the development of the ventral ‘newborn’ SETOA owes much to the ‘older’ and well-established endoscopic endonasal approach (EEA), which has ‘paved the way.’ However, these are distinct techniques with different targets and anatomical structures to reconstruct. Indubitably, thanks to the continuous refinements of the instrumentation, materials and reconstruction techniques [49, 50], especially with the introduction of the nasoseptal flap [51, 52], the incidence of postoperative CSF leak following EEA has evolved over the years from tremendously high rates reported in preliminary series down to nearly 5% in some expert centers [53]. As matter of facts, reconstruction of the lateral orbital wall during SETOA shares some principles and techniques: watertight closure to prevent CSF leak, reduction of the dead space to avoid the risk of enophthalmos but being careful to avoid overpacking and the consequent exophthalmos and visual disturbance. However, the minimal tissue disruption which represents one of the main goals and advantages during the creation of the transorbital corridor, requires a simple and basic reconstruction at the end of the procedure without the need for a vascularized flap. Materials commonly used include dural substitute, free graft of autologous fat, free graft of autologous fascia lata and fibrin glue, adopted in isolated or variously combined manner, as single or multilayers. Generally, in presence of small bony removal, if a dural defect and CFS leak are detected, a free fat graft and fibrin glue are sufficient; conversely, in presence of a larger bony removal, with detection of dural defect and CSF leak, a multilayer reconstruction, including free fat graft and dural substitute or fascia lata, is advisable. No cases of CSF leak are reported after resection of extradural lesions, and rates are very low, 3.05% (n.=5/164) and 6.25% (n.=1/16) for intradural extra-axial and intra-axial lesions, respectively. In detail, among the intradural extra-axial lesions, CSF leak only occurred in meningiomas (n.=5/149), especially those ones at most anterior localization, such as spheno-orbital (n.=3/89, 3.37%) and sphenoid wing (n.=2/45, 4.4%) meningiomas. In these cases, the reconstruction performed was the double-button multilayers technique in all three spheno-orbital meningiomas, with free fat graft and fascia lata or dural substitute intra- and extra-durally placed; in two cases CSF leak resolved with lumbar drainage for few days, while one case required surgical repair. In the two patients with sphenoid wing meningiomas the reconstruction was performed with dural substitute and fat graft; both cases resolved by placing lumbar drainage. From a recent meta-analysis and systematic review on surgical techniques and outcome for spheno-orbital meningiomas [54], in which the extended pterional resulted the workhorse approach (adopted in 97.3% of cases), postoperative CSF leak was reported in 5% of cases. The reconstruction technique was almost heterogeneously distributed: some authors (18%) repaired the dural defect with free graft of fascia, others (16%) with pericranium, whereas for the bony defect, some authors used the titanium mesh (37%), others (29%) the inner calvaria graft or polymethylmethacrylate (26%).

Furthermore, the transorbital pathway presents several intrinsic anatomical advantages when compared to the endonasal route: whereas the EEA uses the natural empty cavities of the nose and sphenoid sinus to reach midline skull base, the SETOA crosses the orbit which is filled by the eyeball which function as a vascularized tissue layer that provides a natural barrier which blocks the CSF leak. In addition, the main issue during the reconstruction phase of EEA is related to the irregular shape of both bony and dural defects and the locations of the optic nerves, internal carotid arteries, and the neurovascular structures of cavernous sinus surrounding them; conversely the bony defect resulting by drilling of lateral orbital wall during SETOA is more regular, roughly trapezoidal in shape, thus the reconstruction materials fits the bony defect more easily. Moreover, the transorbital approach does not work against gravity, as the endonasal approach does, which simplifies reconstruction and further reduces the risk of CSF leakage Finally, the use of preoperative lumbar spinal drainage to decrease the risk of CSF leak intra and postoperatively has been abandoned during EEA by most authors as it has non demonstrated to provide benefits and is not adopted during transorbital approaches [55].

### Limitation of the study

The study has several key limitations. First, the retrospective design of all included studies and the heterogeneity of the data represent significant constraints. Although we did not exclude any prospective or randomized series from our search, the relative novelty of the endoscopic transorbital approach meant that our literature search identified only case reports and retrospective case series.

Most cohorts presented marked disparities in the number of cases, with small sample sizes being a common issue. Significant data variability was also observed among extradural, extra-axial, and intra-axial lesions. As is typical with novel surgical techniques, further studies with larger sample sizes and standardized methodologies are needed to better elucidate advantages, limitations and potential complications of this approach.

The lack of standardized protocols for reconstruction across studies is another critical limitation, as it complicates comparisons and reduces the generalizability of the findings. This lack of standardized reporting reduces the ability to draw definitive conclusions about its impact.

Key clinical variables were inconsistently reported across the included studies, further limiting the scope of our analysis. For instance, the size of the lesion, a factor that may significantly influence outcomes, was not consistently documented and could not be incorporated into our pooled analysis.

The involvement of the orbital wall, including extension or erosion, was another parameter inconsistently documented, despite its potential relevance to postoperative outcomes. Cosmetic outcomes, an important consideration in evaluating endoscopic transorbital approaches, were sporadically reported. This highlights the challenges of assessing and standardizing cosmetic outcomes in the context of novel surgical techniques.

## Conclusion

One of the goal and advantages of the transorbital endoscopic approach is to create a surgical corridor through minimal tissue disruption: small skin incision, periosteal/periorbital dissection, small bone removal; as consequence, a simple and basic reconstruction is required at the end of the procedure without the need for a vascularized flap. Nevertheless, it should be meticulously performed to prevent postoperative complications, first CSF leak which could potentially account for severe morbidity. In conclusion, the SETOA has demonstrated to be a safe approach from this point of view, with a very low rate of CSF leak after addressing intradural pathologies affecting the anterior and middle skull base. However, while the available data suggest a low incidence of CSF leak, variability across studies and inherent limitations prevent definitive recommendations regarding the technique’s overall safety and effectiveness in preventing postoperative CSF leakage from a clinical standpoint.

## Electronic supplementary material

Below is the link to the electronic supplementary material.

Supplementary Material 1

## Data Availability

No datasets were generated or analysed during the current study.

## Abbreviations

SETOA: Superior Eyelid Trans-Orbital Approach
CSF: Cerebrospinal fluid
EEA: Endoscopic Endonasal Approach
