# Correction to: Surgical risk of CSF leakage following endoscopic transorbital approach for anterior and middle skull base pathologies: a systematic review and meta-analysis

**DOI:** 10.1007/s10143-025-03461-w

**Published:** 2025-03-18

**Authors:** Sergio Corvino, Jacopo Berardinelli, Giuseppe Corazzelli, Roberto Altieri, Iacopo Dallan, Francesco Corrivetti, Matteo de Notaris

**Affiliations:** 1 Department of Neurosciences, Reproductive and Odontostomatological Sciences, School of Medicine, Neurosurgical Clinic, University of Naples “Federico II”, 80131 Naples, Italy; 2 Multidisciplinary Department of Medical-Surgical and Dental Specialties, University of Campania “Luigi Vanvitelli”, Naples, Italy; 3 Skull-Base and Rhino-Orbital Surgery Unit, Azienda Ospedaliero-Universitaria Pisana, Pisa, Italy; 4 Department of Neurosurgery, A.O.U. “San Giovanni di Dio e Ruggi d’Aragona”, Salerno, Italy; 5 Department of Neurosciences, Reproductive and Odontostomatological Sciences, Division of Neurosurgery, University of Naples Federico II, 80131 Naples, Italy

**Correction to: Neurosurgical Review**

10.1007/s10143-025-03426-z

The authors regret that there are some errors in the original published article.

The errors and corrections are listed below.

In the **Results** section, paragraph *CSF leak rate*:

- Line 220: the term **“meningioma”** should be added to the following (n.=2/45; 4.44%); *(..sphenoid wing (n.=2/45; 4.44%)****meningioma****and one following an endoscopic amygdalohippocampectomy.)*
- Line 227: The percentage should be **3.05%** instead of **2.2%**, and **6.25%** instead of **6.3%***(..0%*, ***3.05%****and****6.25%***, *respectively)*
- Table 3, under Rate, it should be **3.35%** instead of 10.07%.

In the **Discussion** section, paragraph *CSF leak rate*:

- Line 318–319: The percentage should be **3.05% (n.=5/164)** instead of **2.2% (n.=5/227)**;
- Line 320–321: It should be **(n.=5/149)**, instead of **(n.=5/206)**;
- Line 321–322: It should be **(n.=3/89, 3.37%)** instead of **(n.=3/106, 2.83%)**.

The original article has been corrected.

