# Supplementary material for: Surgical risk of CSF leakage following endoscopic transorbital approach for anterior and middle skull base pathologies: a systematic review and meta-analysis
Source: Neurosurg Rev. 2025 Mar 6;48(1):282. doi: 10.1007/s10143-025-03426-z (PMC11882707; doi:10.1007/s10143-025-03426-z)
Supplement: Supplementary file 1 — Supplementary Material 1 [file 10143_2025_3426_MOESM1_ESM.docx]

**The Endoscopic Transorbital Approach and CSF Leak: Surgical Risk following Anterior and Middle Skull Base Surgery – A Systematic Review**

Sergio Corvino, MD^1^; Jacopo Berardinelli, MD^1^; Giuseppe Corazzelli, MD^1^; Roberto Altieri, MD, PhD^2^; Iacopo Dallan, MD, PhD^3^; Matteo de Notaris, MD, PhD^4^.

^1^Department of Neurosciences, Reproductive and Odontostomatological Sciences, Neurosurgical Clinic; School of Medicine, University of Naples “Federico II”, 80131 Naples, Italy

^2^Multidisciplinary Department of Medical-Surgical and Dental Specialties, University of Campania "Luigi Vanvitelli", Naples, Italy.

^3^Skull-Base and Rhino-orbital Surgery Unit, Azienda Ospedaliero-Universitaria Pisana, Pisa, Italy;

^4^Department of Neurosurgery, A.O.U. “San Giovanni di Dio e Ruggi d’Aragona”, Salerno, Italy

Corresponding Author:

Jacopo Berardinelli, MD

Department of Neurosciences, Reproductive and Odontostomatological Sciences,

Division of Neurosurgery

University of Naples Federico II

80131, Naples, Italy

Email: [iacopobe96@gmail.com](mailto:iacopobe96@gmail.com)

**Search strategy**

**EMBASE**

('cerebrospinal fluid rhinorrhea'/syn OR 'liquorrhea' OR (csf AND leak) OR 'complication'/syn OR 'outcome assessment'/syn) AND ('transorbital approach'/syn OR 'endoscopic transorbital approach'/syn OR (transorbital AND surgery) OR transorbital) AND ('brain tumor'/syn OR 'intradural tumor'/syn OR 'intra axial pathology' OR (intra AND axial AND ('pathology'/exp OR pathology)) OR 'extradural tumor' OR (extradural AND ('tumor'/exp OR tumor)) OR (extra AND axial AND tumor) OR 'skull base'/syn OR 'skull base tumor'/syn OR 'meningioma'/syn OR 'trigeminal schwannoma'/syn OR 'glioma'/syn OR 'brain metastasis'/syn) AND ([article]/lim OR [article in press]/lim OR [data papers]/lim) AND [english]/lim AND [2012-2024]/py

**MEDLINE**

(("cerebrospinal fluid leak"[MeSH Terms] OR ("cerebrospinal"[All Fields] AND "fluid"[All Fields] AND "leak"[All Fields]) OR "cerebrospinal fluid leak"[All Fields] OR "Cerebrospinal Fluid Rhinorrhea"[MeSH Terms] OR ("cerebrospinal fluid rhinorrhoea"[All Fields] OR "Cerebrospinal Fluid Rhinorrhea"[MeSH Terms] OR ("cerebrospinal"[All Fields] AND "fluid"[All Fields] AND "rhinorrhea"[All Fields]) OR "Cerebrospinal Fluid Rhinorrhea"[All Fields]) OR "liquorrhea"[All Fields] OR ("csf"[All Fields] AND "leak"[All Fields]) OR ("outcome assessment, health care"[MeSH Terms] OR ("outcome"[All Fields] AND "assessment"[All Fields] AND "health"[All Fields] AND "care"[All Fields]) OR "health care outcome assessment"[All Fields] OR ("outcome"[All Fields] AND "assessment"[All Fields]) OR "outcome assessment"[All Fields])) AND ((("transorbital"[All Fields] OR "transorbitally"[All Fields]) AND ("approach"[All Fields] OR "approach s"[All Fields] OR "approachability"[All Fields] OR "approachable"[All Fields] OR "approache"[All Fields] OR "approached"[All Fields] OR "approaches"[All Fields] OR "approaching"[All Fields] OR "approachs"[All Fields])) OR (("endoscope s"[All Fields] OR "endoscoped"[All Fields] OR "endoscopes"[MeSH Terms] OR "endoscopes"[All Fields] OR "endoscope"[All Fields] OR "endoscopical"[All Fields] OR "endoscopically"[All Fields] OR "endoscopy"[MeSH Terms] OR "endoscopy"[All Fields] OR "endoscopic"[All Fields]) AND ("transorbital"[All Fields] OR "transorbitally"[All Fields])) OR (("endoscope s"[All Fields] OR "endoscoped"[All Fields] OR "endoscopes"[MeSH Terms] OR "endoscopes"[All Fields] OR "endoscope"[All Fields] OR "endoscopical"[All Fields] OR "endoscopically"[All Fields] OR "endoscopy"[MeSH Terms] OR "endoscopy"[All Fields] OR "endoscopic"[All Fields]) AND ("transorbital"[All Fields] OR "transorbitally"[All Fields]) AND ("approach"[All Fields] OR "approach s"[All Fields] OR "approachability"[All Fields] OR "approachable"[All Fields] OR "approache"[All Fields] OR "approached"[All Fields] OR "approaches"[All Fields] OR "approaching"[All Fields] OR "approachs"[All Fields])) OR (("transorbital"[All Fields] OR "transorbitally"[All Fields]) AND ("surgery"[MeSH Subheading] OR "surgery"[All Fields] OR "surgical procedures, operative"[MeSH Terms] OR ("surgical"[All Fields] AND "procedures"[All Fields] AND "operative"[All Fields]) OR "operative surgical procedures"[All Fields] OR "general surgery"[MeSH Terms] OR ("general"[All Fields] AND "surgery"[All Fields]) OR "general surgery"[All Fields] OR "surgery s"[All Fields] OR "surgerys"[All Fields] OR "surgeries"[All Fields])))) AND ((english[Filter]) AND (2012:2024[pdat]))

**Table OR1** Excluded studies

| **Cause for exclusion** | **Author and date of publication** | |
| --- | --- | --- |
| **Transcranial EEA approach either alone or combined without segregation of outcome** | Almeida et al 2017 (1)  Chabot et al 2017 (2)  Dallan et al 2018 (3)  d’Avella et al 2024 (4)  Feller et al 2023 (5)  Jung et al 2022 (6)  Mariniello et al 2024 (7)  Noiphithak et al 2023 (8) | Park et al 2019 (9)  Park et al 2020 (10)  Ulutas et al 2021 (11)  Scholfield et al 2024 (12)  Shapira et al 2022 (13)  Wang et al 2024 (15)  Zoli et al 2023 (16) |
| **Series by the same center** | Carnevale et al 2023 (17)  Dallan et al 2015 (18)  Di Somma et al 2021 (19) | Lee et al 2019 (20)  Lee et al 2022 (21)  Yoo et al 2021 (22) |
| **No Intracranial disease** | Govind et al 2023 (23)  Raza et al 2013 (24)  Kim et al 2024 (25) | Koutourousiou et al 2012 (26)  Zoia et al 2024 (27) |
| **Cadaveric study** | Matano et al 2022 (28)  Gosal et al 2024 (29) | Hong et al 2024 (30) |
| **Operative video** | Noiphithak et al 2022 (32) |  |

**Figure OR1** Pooled analysis and comparison revealed an no differences in postoperative CSF leaks following dural reconstruction with dural substitutes or fascia lata (p=0.47, I^2^=0%) or with implementation of fat free grafts (p=0.17, I^2^=5%)


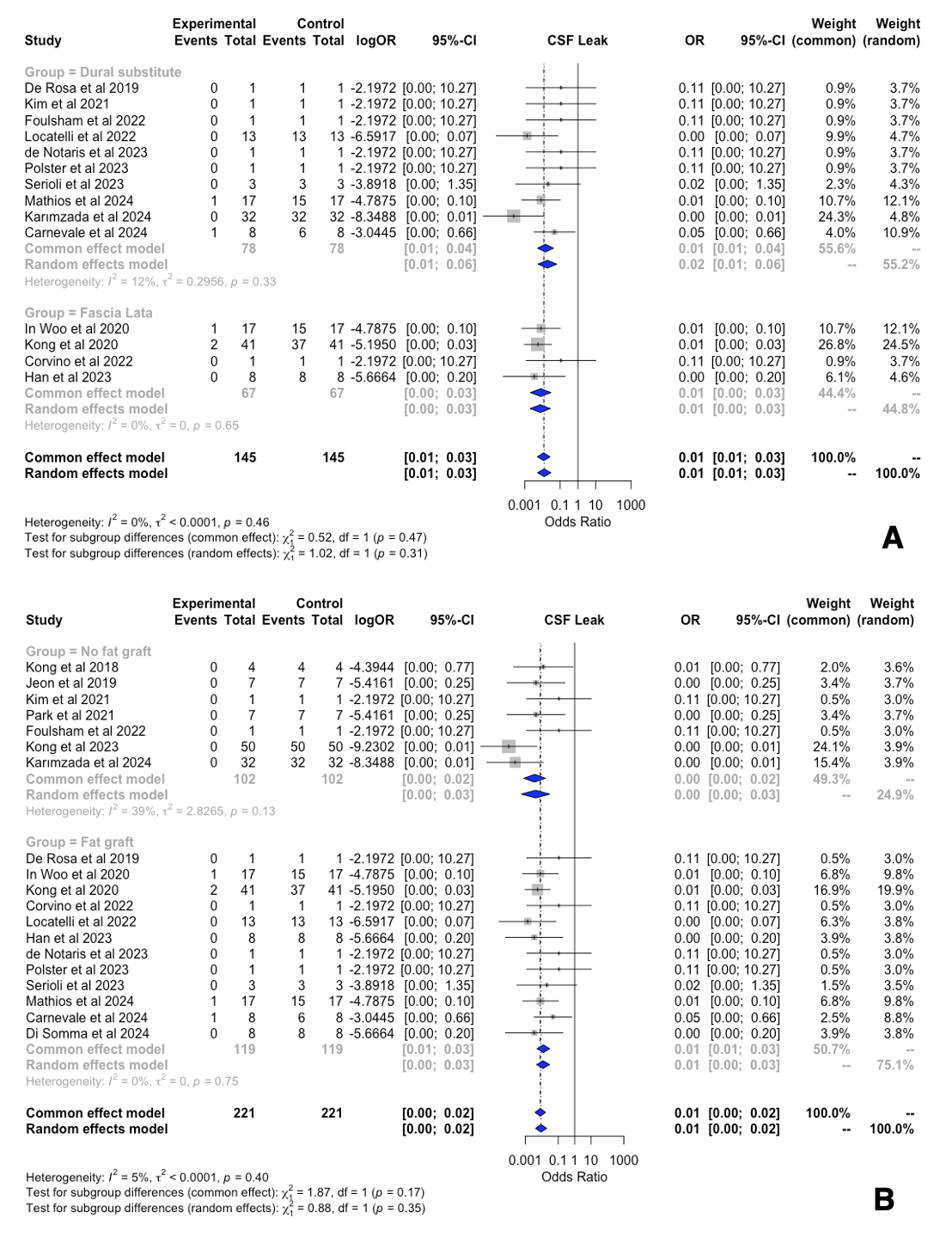


**Figure OR2**ROBINS-I tool to evaluate the Risk of Bias for each included study


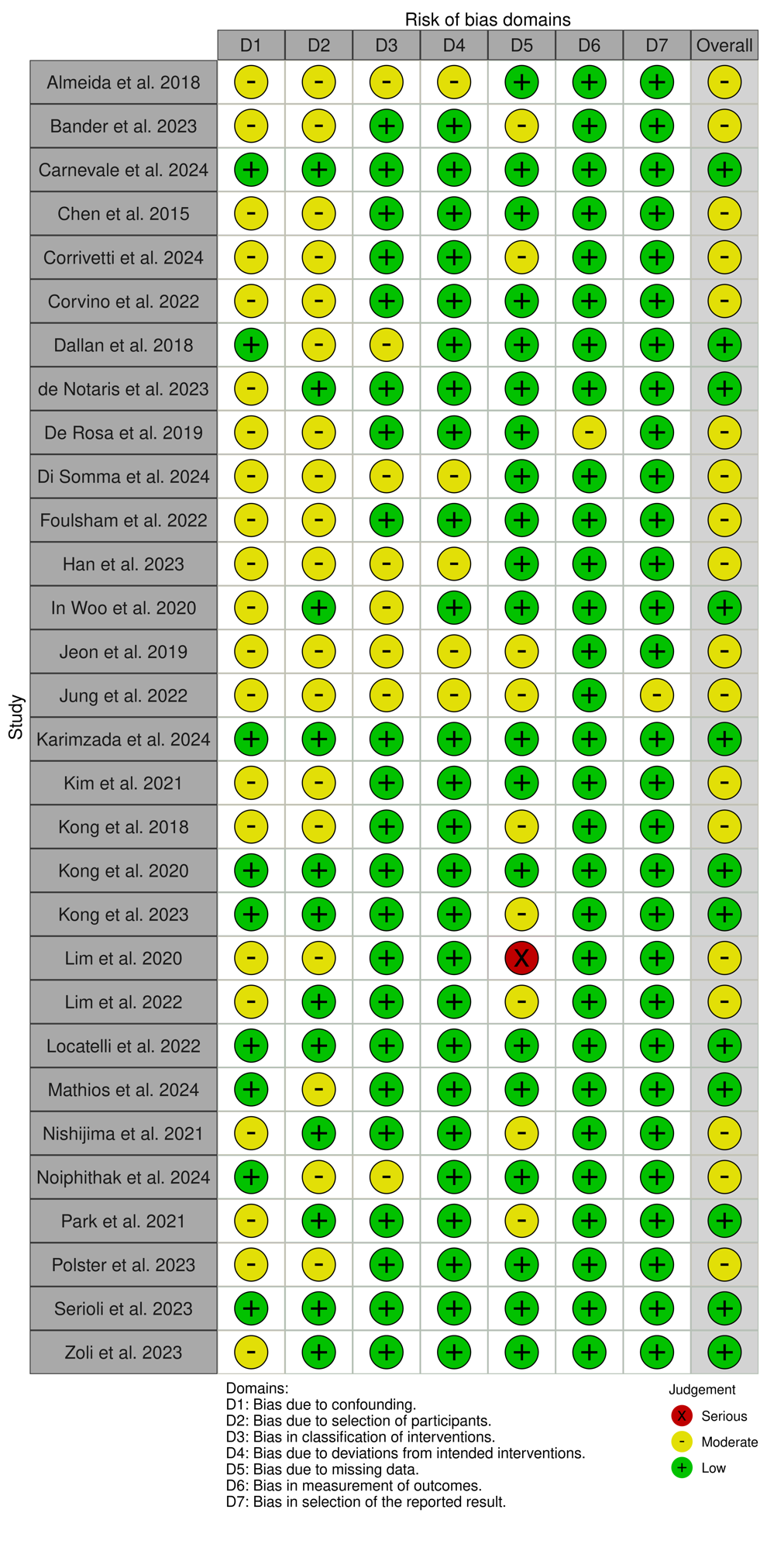


**Figure OR3**Aggregated overall and single domains Risk of Bias for the included studies.


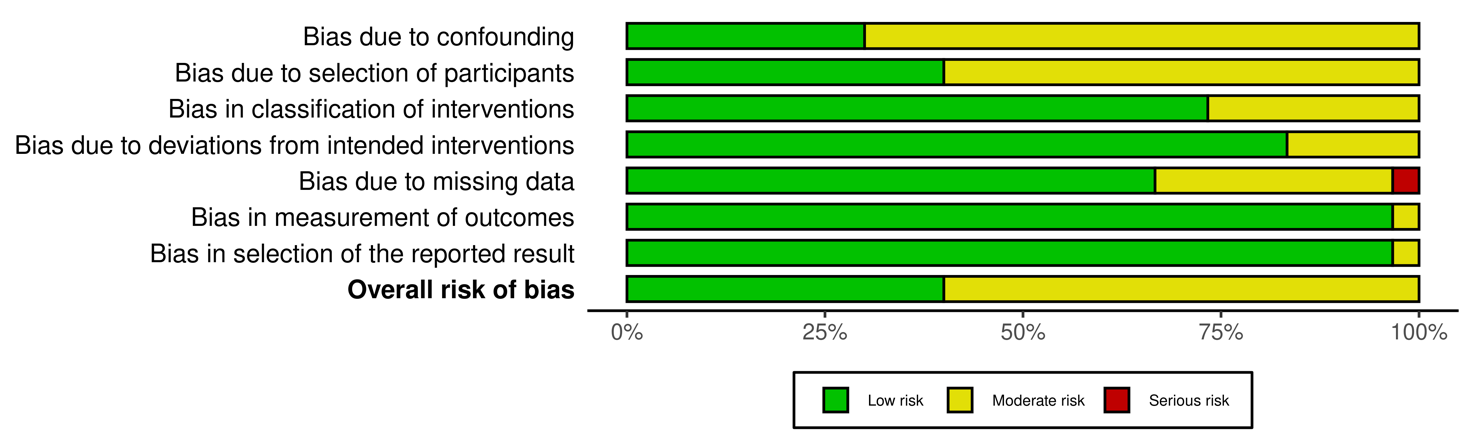


**References**

1. Almeida JP, Omay SB, Shetty SR, Chen YN, Ruiz-Treviño AS, Liang B, et al. Transorbital endoscopic eyelid approach for resection of sphenoorbital meningiomas with predominant hyperostosis: report of 2 cases. J Neurosurg. 2018;128(6):1885-95.

2. Chabot JD, Gardner PA, Stefko ST, Zwagerman NT, Fernandez-Miranda JC. Lateral Orbitotomy Approach for Lesions Involving the Middle Fossa: A Retrospective Review of Thirteen Patients. Neurosurgery. 2017;80(2):309-22.

3. Dallan I, Sellari-Franceschini S, Turri-Zanoni M, de Notaris M, Fiacchini G, Fiorini FR, et al. Endoscopic Transorbital Superior Eyelid Approach for the Management of Selected Spheno-orbital Meningiomas: Preliminary Experience. Oper Neurosurg (Hagerstown). 2018;14(3):243-51.

4. d'Avella E, Somma T, Fabozzi GL, Committeri U, Romano A, Cappabianca P, et al. Endoscopic transorbital and transcranial multiportal resection of a sphenoorbital meningiomas with custom bone 3D printing reconstruction: Case report. Head Neck. 2024;46(2):E18-E25.

5. Feller C, Martinez Del Campo E, Eraky AM, Montoure A, Maloley L, Harrison G, et al. Transorbital approach for resection of intracranial skull base lesions: Outcomes and complications. Interdisciplinary Neurosurgery. 2023;32:101747.

6. Jung IH, Yoo J, Choi S, Lim SH, Ko J, Roh TH, et al. Endoscopic transorbital approach to the cavernous sinus: Cadaveric anatomy study and clinical application (Front Oncol. 2022;12:962598.

7. Mariniello G, Corvino S, Corazzelli G, de Divitiis O, Fusco G, Iuliano A, et al. Spheno-Orbital Meningiomas: The Rationale behind the Decision-Making Process of Treatment Strategy. Cancers (Basel). 2024;16(11).

8. Noiphithak R, Yanez-Siller JC, Nimmannitya P, Yodwisithsak P. Optimizing Management of Cavernous Sinus Meningiomas: A Novel Strategy Integrating Endoscopic Endonasal and Transorbital Surgery with Adjuvant Stereotactic Radiotherapy. World Neurosurg. 2024.

9. Park HH, Yoo J, Yun IS, Hong CK. Comparative Analysis of Endoscopic Transorbital Approach and Extended Mini-Pterional Approach for Sphenoid Wing Meningiomas with Osseous Involvement: Preliminary Surgical Results. World Neurosurg. 2020;139:e1-e12.

10. Park HH, Hong SD, Kim YH, Hong CK, Woo KI, Yun IS, et al. Endoscopic transorbital and endonasal approach for trigeminal schwannomas: a retrospective multicenter analysis (KOSEN-005). J Neurosurg. 2020;133(2):467-76.

11. Ulutas M, Çinar K, Dogan I, Secer M, Isik S, Aksoy K. Lateral transorbital approach: an alternative microsurgical route for supratentorial cerebral aneurysms. J Neurosurg. 2021;134(1):72-83.

12. Scholfield DW, Levyn H, Tabar VS, Ganly I, Della Rocca D, Cohen MA. The medial transorbital approach in cranioendoscopic skull base tumor resections for locally advanced tumors. J Clin Neurosci. 2024;119:198-204.

13. Shapira Y, Juniat V, Dave T, Hussain A, McNeely D, Watanabe A, et al. Orbito-cranial schwannoma-a multicentre experience. Eye (Lond). 2023;37(1):48-53.

14. Wang H, Luo L, Ye Z, Li W, Chen C, Ba Y, et al. Clipping of anterior communicating artery aneurysms in the early post-rupture stage via transorbital keyhole approach--Chinese neurosurgical experience. Br J Neurosurg. 2015;29(5):644-9.

15. Wang Q, Xu X, Ouyang S, Chen J, Song Z, Lou J, et al. Exposure of the Cavernous Sinus via the Endoscopic Transorbital and Endoscopic Endonasal Approaches: A Comparative Study. World Neurosurg. 2024;181:e1047-e58.

16. Zoli M, Sollini G, Rustici A, Guaraldi F, Asioli S, Altavilla MV, et al. Endoscopic Transorbital Approach for Spheno-Orbital Tumors: Case Series and Systematic Review of Literature. World Neurosurg. 2023.

17. Carnevale JA, Rosen KU, Chae JK, Pandey A, Bander ED, Godfrey K, et al. The Endoscopic Lateral Transorbital Approach for the Removal of Select Sphenoid Wing and Middle Fossa Meningiomas. Surgical Technique and Short-Term Outcomes. Oper Neurosurg (Hagerstown). 2024;26(2):165-72.

18. Dallan I, Castelnuovo P, Locatelli D, Turri-Zanoni M, AlQahtani A, Battaglia P, et al. Multiportal Combined Transorbital Transnasal Endoscopic Approach for the Management of Selected Skull Base Lesions: Preliminary Experience. World Neurosurg. 2015;84(1):97-107.

19. Di Somma A, Langdon C, de Notaris M, Reyes L, Ortiz-Perez S, Alobid I, et al. Combined and simultaneous endoscopic endonasal and transorbital surgery for a Meckel's cave schwannoma: technical nuances of a mini-invasive, multiportal approach. J Neurosurg. 2021;134(6):1836-45.

20. Lee MH, Hong SD, Woo KI, Kim YD, Choi JW, Seol HJ, et al. Endoscopic Endonasal Versus Transorbital Surgery for Middle Cranial Fossa Tumors: Comparison of Clinical Outcomes Based on Surgical Corridors. World Neurosurg. 2019;122:e1491-e504.

21. Lee WJ, Hong SD, Woo KI, Seol HJ, Choi JW, Lee JI, et al. Combined endoscopic endonasal and transorbital multiportal approach for complex skull base lesions involving multiple compartments. Acta Neurochir (Wien). 2022;164(7):1911-22.

22. Yoo J, Park HH, Yun IS, Hong CK. Clinical applications of the endoscopic transorbital approach for various lesions. Acta Neurochir (Wien). 2021;163(8):2269-77.

23. Govind A, Demirel S, Lee K, Amundson M, Bell RB, Dierks E. Predictors of Intraoperative Difficulty and Postoperative Examination Abnormalities in 164 Orbital Operations. J Oral Maxillofac Surg. 2023;81(11):1360-71.

24. Raza SM, Quinones-Hinojosa A, Lim M, Boahene KD. The transconjunctival transorbital approach: a keyhole approach to the midline anterior skull base. World Neurosurg. 2013;80(6):864-71.

25. Kim JH, Hong CK, Shin HJ, Kong DS. Feasibility and efficacy of endoscopic transorbital optic canal decompression for meningiomas causing compressive optic neuropathy. J Neurosurg. 2024;140(2):412-9.

26. Koutourousiou M, Gardner PA, Stefko ST, Paluzzi A, Fernandez-Miranda JC, Snyderman CH, et al. Combined endoscopic endonasal transorbital approach with transconjunctival-medial orbitotomy for excisional biopsy of the optic nerve: technical note. J Neurol Surg Rep. 2012;73(1):52-6.

27. Zoia C, Maiorano E, Borromeo S, Mantovani G, Spena G, Pagella F. Endoscopic approaches to the orbit: Transnasal and transorbital, a retrospective case series. Brain Spine. 2024;4:102770.

28. Matano F, Passeri T, Abbritti R, Camara B, Mastantuoni C, Noya C, et al. Eyebrow incision with a crescent-shaped orbital rim craniotomy for microscopic and endoscopic transorbital approach to the anterior and middle cranial fossa: A cadaveric study and case presentation. Brain Spine. 2022;2:100891.

29. Gosal JS, Bhuskute GS, Alsavaf MB, Abouammo MD, Manjila S, Alwabili M, et al. Comparison of endoscopic multiport approaches to the petrous apex: contralateral transmaxillary versus contralateral medial transorbital corridor. J Neurosurg. 2024;141(6):1494-504.

30. Hong CK, Mosteiro A, Kong DS, Tafuto R, Codes M, Ferres A, et al. Endoscopic transorbital approach to the petrous apex: is orbital rim removal worthwhile for the exposure? An anatomical study with illustrative case. J Neurosurg. 2024;141(6):1595-603.

31. Piper K, Saez-Alegre M, George Z, Srivastava A, Felbaum DR, Jean WC. Transorbital Surgical Corridor: An Anatomic Analysis of Ocular Globe Retraction and the Associated Exposure for the Transpalpebral Orbital Rim Preserving Endoscopic Orbitotomy (TORPEDO) Approach. Oper Neurosurg (Hagerstown). 2024;26(2):196-202.

32. Noiphithak R, Yanez-Siller JC, Nimmannitya P. Transorbital Approach for Olfactory Groove Meningioma. World Neurosurg. 2022;162:66.
